# Aging Trajectory Analysis of Asphalt: Differential Regulation of UV Aging Processes by Anti-Aging Agents with Varied Mechanisms

**DOI:** 10.3390/ma19132740

**Published:** 2026-06-26

**Authors:** Hui Wang, Ping Li, Le Yang, Xingzhen Zang, Longyuan Su, Jingzhuo Zhao

**Affiliations:** 1 School of Civil and Hydraulic Engineering, Lanzhou University of Technology, Lanzhou 730050, China; huiwang1190@sina.cn (H.W.);; 2 Gansu Province Transportation Planning Survey & Design Institute Co., Ltd., Lanzhou 730030, China; 3 Dingxi Highway Development Center of Gansu Province, Dingxi 743099, China

**Keywords:** asphalt, ultraviolet (UV) aging, anti-aging agents, principal component analysis (PCA), aging trajectory

## Abstract

**Highlights:**

- Decoupling aging pathways to classify anti-UV agents’ effects using dynamic analysis.
- Trajectory analysis was applied to monitor UV aging evolution in asphalt dynamically.
- Combining physical, rheological, and chemical tests with PCA for full aging evaluation.
- Modified asphalts exhibit reduced overall UV ageing, especially layered silicate.

**Abstract:**

In this study, four types of anti-ultraviolet aging agents—layered double hydroxides (LDHs), organic montmorillonite (OMMT), titanium dioxide (TiO_2_), and ultraviolet absorber (UV326)—were employed to modify asphalt. The modified asphalt samples underwent Rolling Thin Film Oven Test (RTFOT) and xenon-lamp aging treatments, and we examined the evolution of their physical properties, rheological performance, and chemical composition. A principal component analysis (PCA) model built on representativeness, discriminative power, and non-redundancy reduced the multidimensional data to two principal components, which together captured 87.540% of the total variance. The dynamic principal component trajectories, plotted from the reduced-dimension data for the unaged–short-term-aged–xenon-lamp-aged process, revealed that anti-aging agents sharing the same protection mechanism led to comparable rates of high- and low-temperature performance deterioration during xenon-lamp aging, whereas agents with different mechanisms resulted in distinctly different patterns of performance deterioration. In the critical xenon-lamp aging stage, the neat asphalt exhibited a trajectory vector change of ΔPC1 = 0.92 and ΔPC2 = 1.25, corresponding to an angle of 54°, reflecting a low-temperature degradation. By contrast, the physical shielding agents LDHs and OMMT produced much steeper trajectories with angles of approximately −80°, where ΔPC2 values rose to as high as 3.67 and 2.19 respectively despite modest reductions in overall aging. The reflective agent TiO_2_ showed a more moderate angle of 84°, with ΔPC1 and ΔPC2 values of 0.16 and 1.45, indicating a slight retardation of high-temperature performance loss. Notably, the UV absorber UV326 maintained the same trajectory angle of 56° as the neat asphalt but with reduced magnitudes of ΔPC1 = 0.63 and ΔPC2 = 0.94, suggesting a balanced delay in aging without altering its relative progression. This study proposes a novel analytical framework for mechanism-based clustering analysis and the precise selection of anti-aging agents for asphalt.

## 1. Introduction

As a key binding material in pavements, the long-term durability of asphalt directly governs pavement performance and service life. Among various environmental aging factors, ultraviolet (UV) radiation—due to its short wavelength and high energy—triggers free radical reactions that lead to asphalt hardening, loss of ductility, and deterioration of viscoelastic properties, making UV radiation one of the primary causes of asphalt pavement deterioration [1,2,3]. Consequently, enhancing the resistance of asphalt to UV aging has emerged as a central focus in contemporary road engineering research [4,5,6].

Incorporating ultraviolet anti-aging agents represents an effective strategy for enhancing the weathering resistance of asphalt. Commonly used additives typically function through three primary mechanisms: (1) physical shielding, which utilizes nano-layered structures to block the transmission path of UV radiation [7]; (2) optical reflection, where materials with high refractive indices reflect UV radiation [8,9]; and (3) ultraviolet absorption, wherein the molecular structure dissipates UV energy by converting it into other forms of energy [10,11].

Although numerous types of additives have demonstrated favorable anti-UV aging effects in a wide range of studies, relatively few investigations have systematically compared anti-aging agents with distinct mechanisms of action or elucidated their differences in regulating the asphalt aging process [11,12,13,14]. This is primarily due to the strong interrelationships among the physical, rheological, and chemical compositional characteristics of asphalt [15,16,17,18], which complicates the rational selection of evaluation indices and the avoidance of informational redundancy when assessing the degree of asphalt aging [19,20,21,22]. Furthermore, relying solely on comparisons of performance indicators before and after aging makes it challenging to distinguish whether an anti-aging agent merely slows the aging rate or fundamentally alters the aging pathway [12,23].

**Scheme 1:**
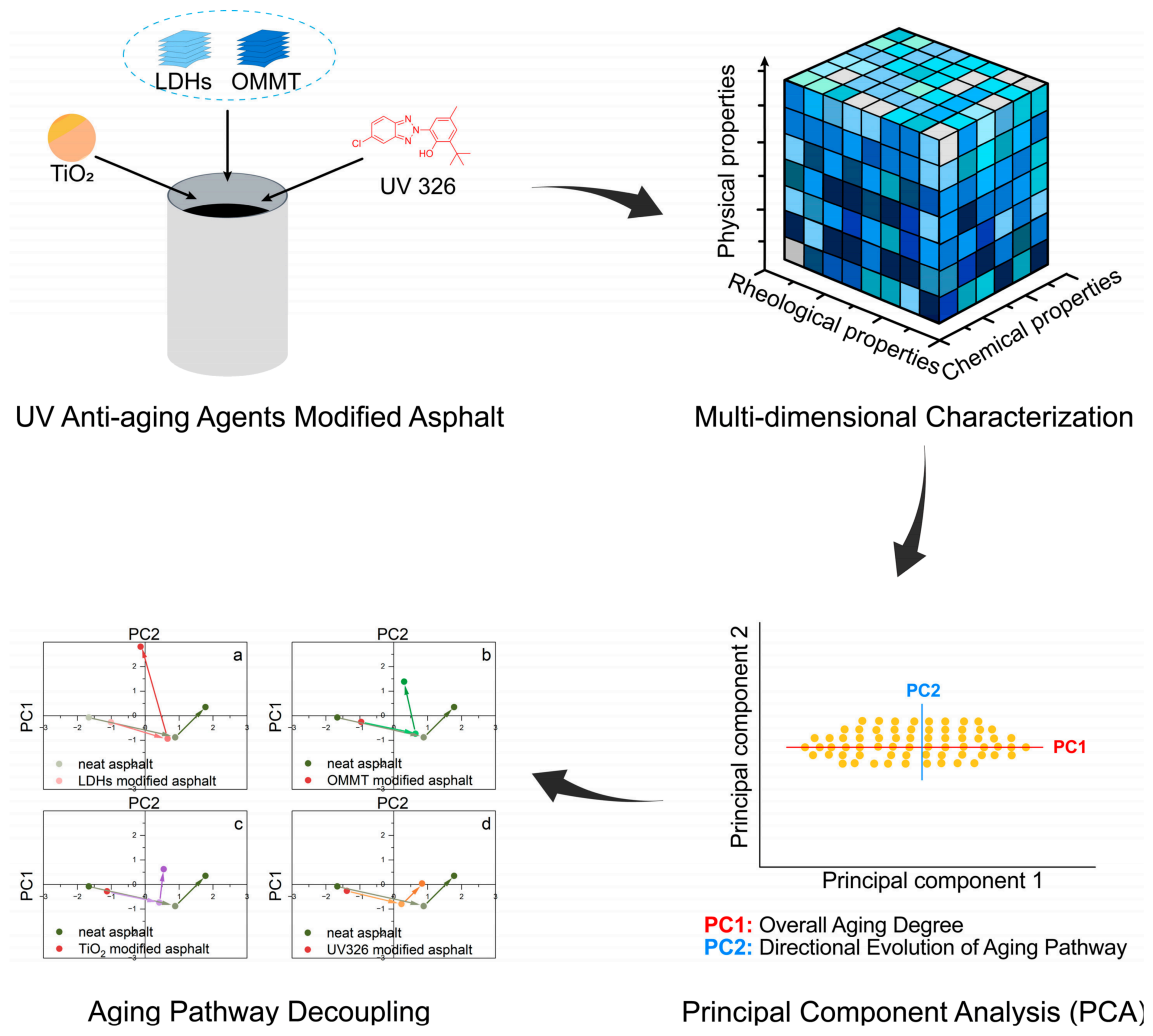
Schematic illustration of the research framework.

To address the aforementioned issues, this study selected four types of anti-UV aging agents based on distinct mechanisms: layered double hydroxides (LDHs) and organic montmorillonite (OMMT) representing physical shielding, titanium dioxide (TiO_2_) for reflective action, and ultraviolet absorber (UV326) for absorption. Each agent was used to modify base asphalt separately. The modified asphalt samples were subjected to xenon-lamp aging tests to simulate UV radiation, and changes in their physical, rheological, and chemical composition characteristics before and after aging were measured. Furthermore, based on these data, a systematic principal component analysis (PCA) model was established using representativeness, discriminative power, and non-redundancy as variable selection criteria. Combined with dynamic aging trajectory analysis, this approach aims to clarify the differential regulatory effects of various anti-aging agents on the relative rates of high- and low-temperature performance deterioration in asphalt (Scheme 1).

This approach establishes a new paradigm for elucidating the explicit relationship between anti-UV aging agents and performance loss during asphalt aging, thereby offering methodological and theoretical support for the targeted selection of anti-aging agents based on specific performance objectives and protective mechanisms. It should be noted that in this study, the term “asphalt” is used to refer exclusively to the bitumen binder, not to the asphalt mixture or pavement composite.

## 2. Materials and Methods

### 2.1. Materials

A 90# Grade A asphalt commonly used in China was selected as the base material, and its properties are summarized in Table 1.

Four commercially available UV anti-aging agents were used in this study, as described below:

LDHs: magnesium–aluminum carbonate-type layered double hydroxides ([Mg_6_Al_2_(OH)_16_CO_3_]·4H_2_O), commercially purchased from Jiangyin Ruifa Chemical Co., Ltd. (Jiangyin, China).

TiO_2_: rutile-phase titanium dioxide with an average particle size of approximately 48 nm, commercially purchased from Guangdong Baisi Chemical Technology Co., Ltd. (Guangzhou, China).

OMMT: organo-montmorillonite modified with dioctadecyldimethylammonium chloride, with an interlayer spacing of about 2.16 nm, commercially purchased from Nanjing XFNANO Materials Tech Co., Ltd. (Nanjing, China).

UV326: 2-(2′-hydroxy-3′-tert-butyl-5′-methylphenyl)-5-chlorobenzotriazole, a pale crystalline powder, commercially purchased from Dongguan Shanyi Plastification Co., Ltd. (Dongguan, China).

### 2.2. Equipment

The morphology and structure of the UV anti-aging agents were characterized using a Shimadzu XRD-7000 X-ray diffractometer (Kyoto, Japan) and a Guoyi Quantum SEM5000 scanning electron microscope (SEM) (Hefei, China).

Short-term asphalt aging was conducted using a CS 325-B Rolling Thin Film Oven manufactured by James Cox & Sons (Colfax, CA, USA). Xenon-lamp aging tests were performed in a Q-Sun Xe-3 HSE xenon test chamber produced by Q-LAB (Westlake, OH, USA).

Penetration, softening point, and ductility of asphalt samples were measured using an 81-PV0103 penetrometer, an 81-PV0144 automatic softening point tester, and an 81-PV10020 ductility tester, all manufactured by CONTROLS (Liscate, Milan, Italy). Viscosity was determined with a DV2T viscometer (BROOKFIELD, Middleboro, MA, USA). Rheological properties were evaluated through Dynamic Shear Rheometry (DSR) and Multiple Stress Creep Recovery (MSCR) tests using an Anton Paar MCR 302 dynamic shear rheometer (Anton Paar, Graz, Austria). Fourier transform infrared (FTIR) spectroscopy was performed on a Nicolet iS50 spectrometer produced by Thermo Fisher Scientific Inc. (Madison, WI, USA), equipped with an Attenuated Total Reflection (ATR) accessory.

### 2.3. Experiments

#### 2.3.1. Characterization of the UV Anti-Aging Agents

X-ray diffraction (XRD) analysis was performed using Cu-Kα radiation with a wavelength of 0.15406 nm. The tube voltage and current were set to 40 kV and 40 mA, respectively. Scanning was conducted at a rate of 2°/min over a 2θ range from 0.5° to 60°. SEM observations employed Au as the coating material, the acceleration voltage was set between 20 and 30 kV.

#### 2.3.2. Preparation of UV Anti-Aging Agent-Modified Asphalt

The base asphalt was heated to a molten, flowable state at 135°C. A 500 g sample was weighed, and 3 wt% of the anti-aging agent was slowly added under mechanical stirring at 1000 rpm, followed by premixing for 15 min to ensure full wetting. The mixture was then transferred to a high-speed shear mixer and sheared at 5000 rpm for 1 h at the corresponding temperature to achieve uniform additive dispersion. The temperature was maintained for an additional 2.5 h to promote dispersion and maturation, yielding the UV anti-aging agent-modified asphalt.

**Figure A1:**
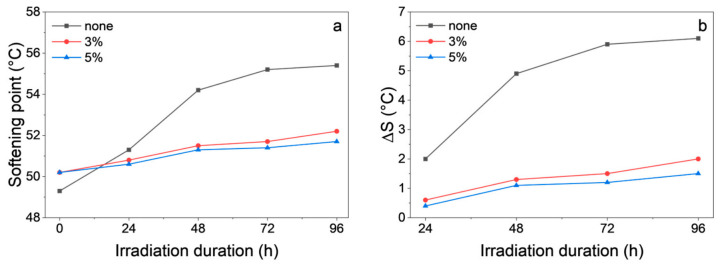
Variation of (**a**) softening point and (**b**) increase in softening point (ΔS) of LDH-modified asphalt with different LDHs contents as a function of xenon lamp aging duration.

**Figure A2:**
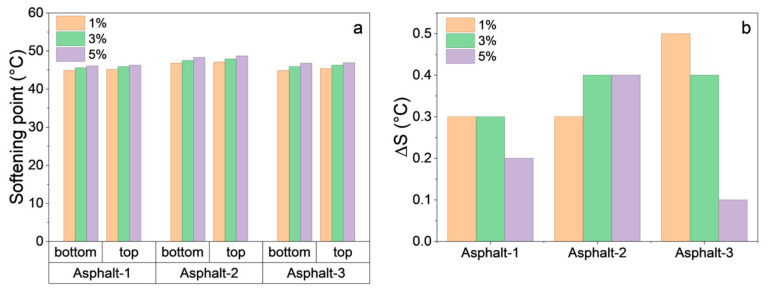
(**a**) Softening points at different positions and (**b**) softening point difference (ΔS) of LDH-modified asphalt specimens.

The additive dosage was uniformly set at 3% by weight of asphalt. This dosage was selected to ensure a fair mechanistic comparison by keeping all additives within their effective-response windows without entering saturation or causing dispersion problems. For LDHs, our preliminary tests showed that both 3% and 5% additions reduced the UV-induced softening point increment to a similar extent, indicating an efficacy plateau near 3% (Figure A1); storage stability was acceptable up to 5% (Figure A2). For OMMT, TiO_2_ and UV326, reported effective dosages typically range from 0.5 to 3 wt% [24], making 3% a representative common baseline.

#### 2.3.3. Accelerated Aging of Asphalt

Short-term aging of asphalt was performed using the Rolling Thin Film Oven Test (RTFOT) in accordance with ASTM D2872-22 [25].

The equivalent exposure duration was determined based on the annual total solar radiation at the Dunhuang exposure site in Gansu Province, China. The annual total solar radiation at this location is approximately 5805.1561 MJ/m^2^. To estimate the ultraviolet portion, a reduction factor of 6% was applied, following the approach reported in the literature [26]. The xenon-lamp aging chamber was operated at 60°C under an irradiation intensity of 180 W/m^2^. Using these values, the required exposure time was calculated as Equation (1). Accordingly, all asphalt specimens subjected to xenon-lamp aging in this study were continuously irradiated for a total duration of 22.39 days.

(1) $$\frac{5805.1561\times 10^{6}\;J/m^{2}\times 6\%}{180\;W/m^{2}}=1.9351\times 10^{6}\;s=22.39\;d$$

All UV anti-aging agent-modified asphalt samples were designated and named according to Table 2.

#### 2.3.4. Asphalt Performance Testing

Softening point, penetration, ductility, and viscosity tests were conducted in accordance with ASTM D36/D36M-14 (2020) [27], ASTM D5/D5M-20 [28], ASTM D113/D113M-17 (2023)e1 [29], and ASTM D4402/D4402M-23 [30], respectively. DSR and MSCR tests were performed following ASTM D8189-19 [31] and ASTM D7405-24 [32], respectively.

#### 2.3.5. FTIR Spectroscopy Testing

A small amount of asphalt was heated and evenly coated onto the surface of the ATR crystal for measurement. The FTIR spectrometer was operated with a scanning range of 500–4000 cm^−1^, a resolution of 4 cm^−1^, and 32 scans.

## 3. Results

### 3.1. Microstructure of UV Anti-Aging Agents

**Figure 1:**
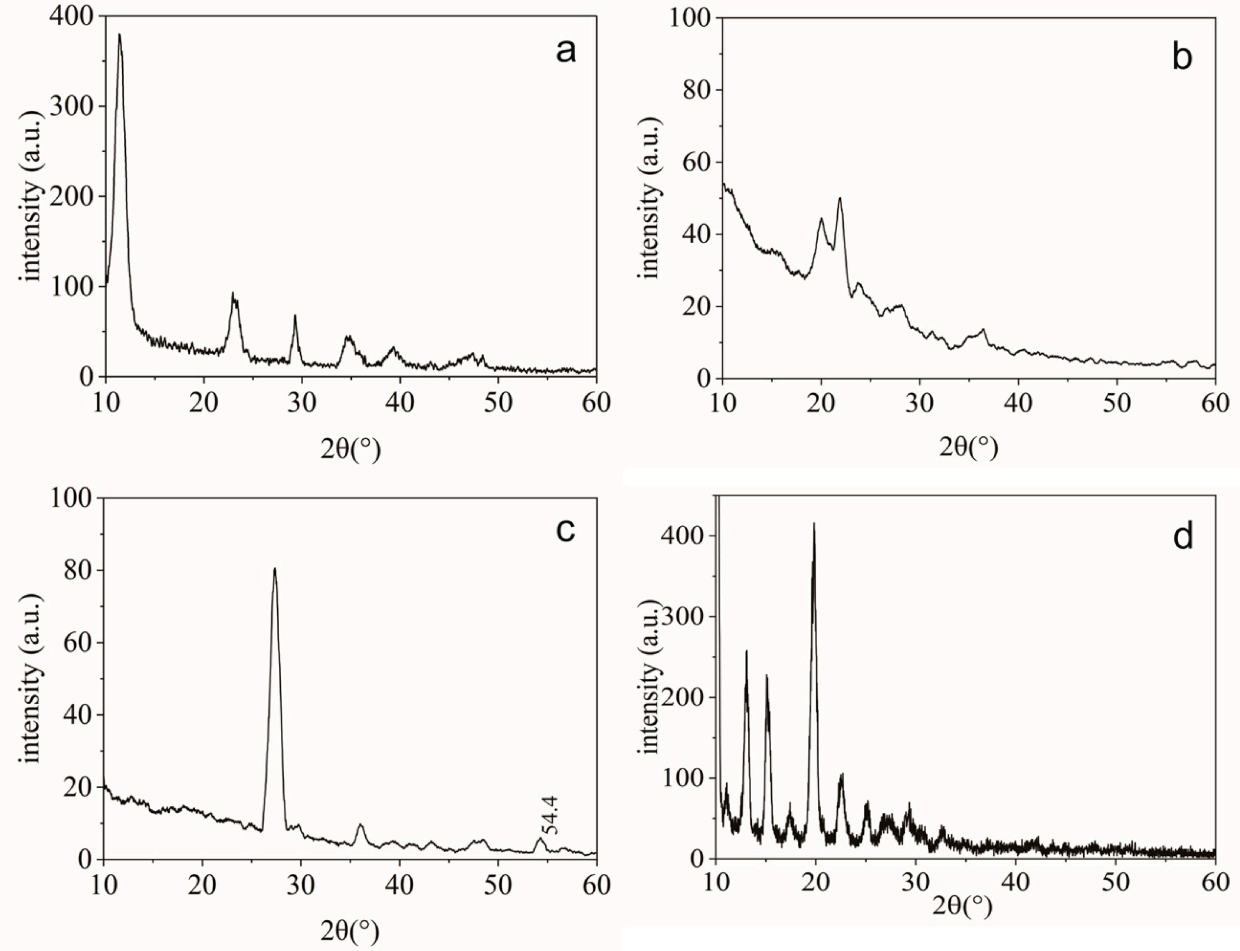
XRD spectrum of (**a**) LDHs, (**b**) OMMT, (**c**) TiO_2_ and (**d**) UV326.

Figure 1 presents the XRD patterns of the various UV anti-aging agents. LDHs exhibit distinct characteristic diffraction peaks at 11.42°, 23.4°, 34.92°, 39.44°, and 47.5°, corresponding to the (003), (006), (009), (015), and (018) crystal planes of hydrotalcite, respectively. These peaks are sharp, symmetric, and of high intensity, indicating an ordered layered stacking structure, well-defined crystallinity, and a high degree of crystalline order in the LDHs. OMMT displays characteristic diffraction peaks of montmorillonite at 20.14°, 35.1°, and 62.32°, which correspond to the (100), (105), and (300) crystal planes, respectively. Nano-TiO_2_ shows characteristic peaks at 27.7°, 36.2°, and 54.4°, attributed to the (110), (101), and (211) planes of the rutile phase of TiO_2_. No characteristic peaks of anatase TiO_2_ are observed in the spectrum, confirming that the nano-TiO_2_ is purely in the rutile phase. UV326 exhibits characteristic diffraction peaks at 13.14°, 15.1°, 19.82°, and 22.46°, suggesting a relatively large interlayer spacing.

**Figure 2:**
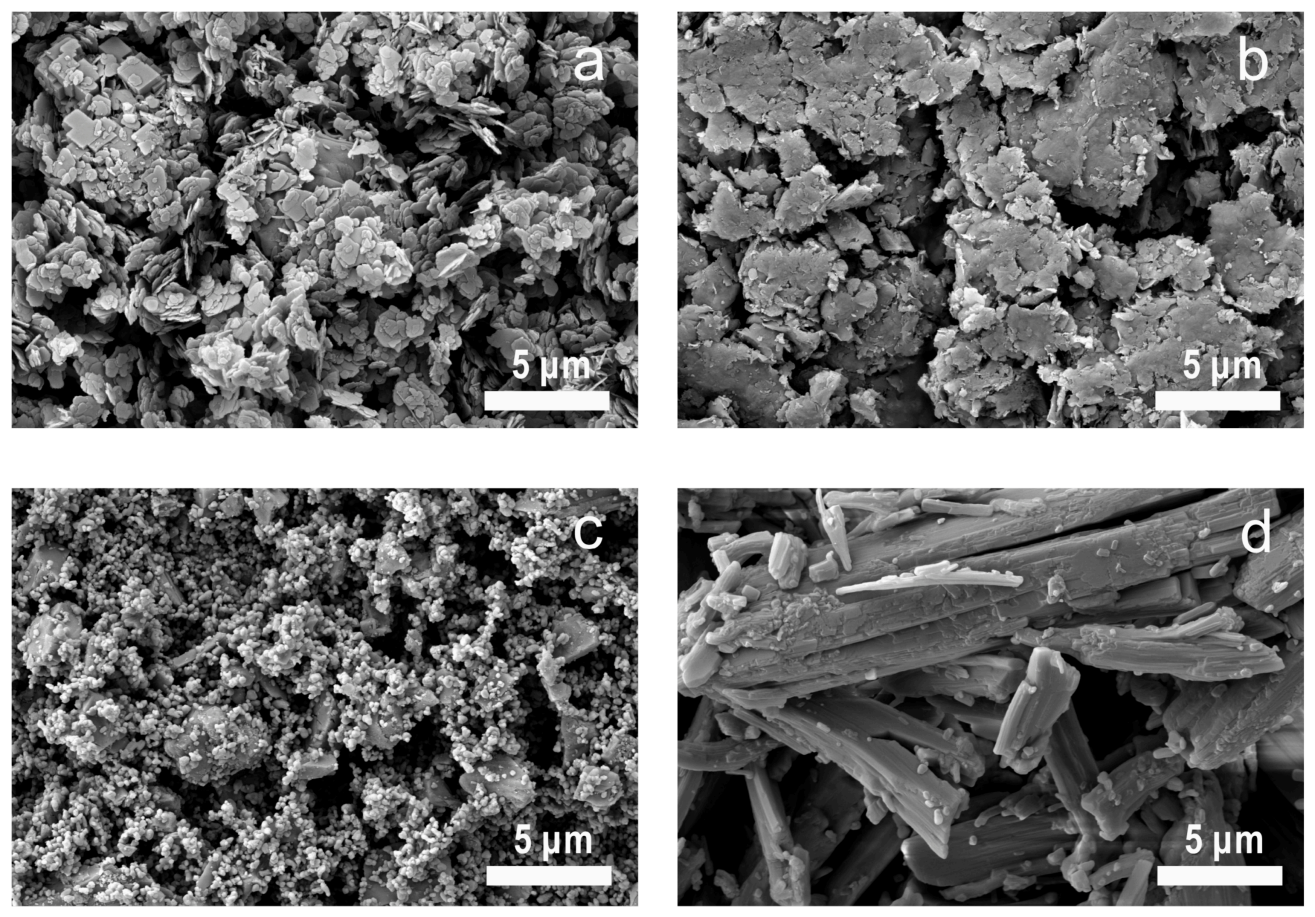
SEM morphology of (**a**) LDHs, (**b**) OMMT, (**c**) TiO_2_ and (**d**) UV326. All images were acquired at a magnification of ×5000.

SEM results (Figure 2) further corroborate the structural analysis described above. Both LDHs and OMMT appear as irregular flakes of varying sizes with evident aggregation. The aggregation in LDHs is attributed to hydrogen bonding and van der Waals forces resulting from the abundant free hydroxyl groups on the surface of their metal layers, whereas in OMMT, it arises from increased intermolecular cohesion due to organic modification. Nano-TiO_2_ exists in a fine particulate form, while UV326 exhibits an ordered and well-defined layered structure.

### 3.2. Evolution of Macroscopic Physical Properties

The incorporation of various UV anti-aging agents leads to an increase in the softening point of asphalt, which can be attributed to the filler-like effect of these powdered additives within the asphalt matrix [33]. Following short-term aging, the softening point of all samples rises further, primarily due to the volatilization of light components, resulting in a higher proportion of resins and asphaltenes. Under the more severe xenon-lamp aging conditions, UV-induced oxidative hardening leads to a further increase in the softening point for all samples.

**Figure 3:**
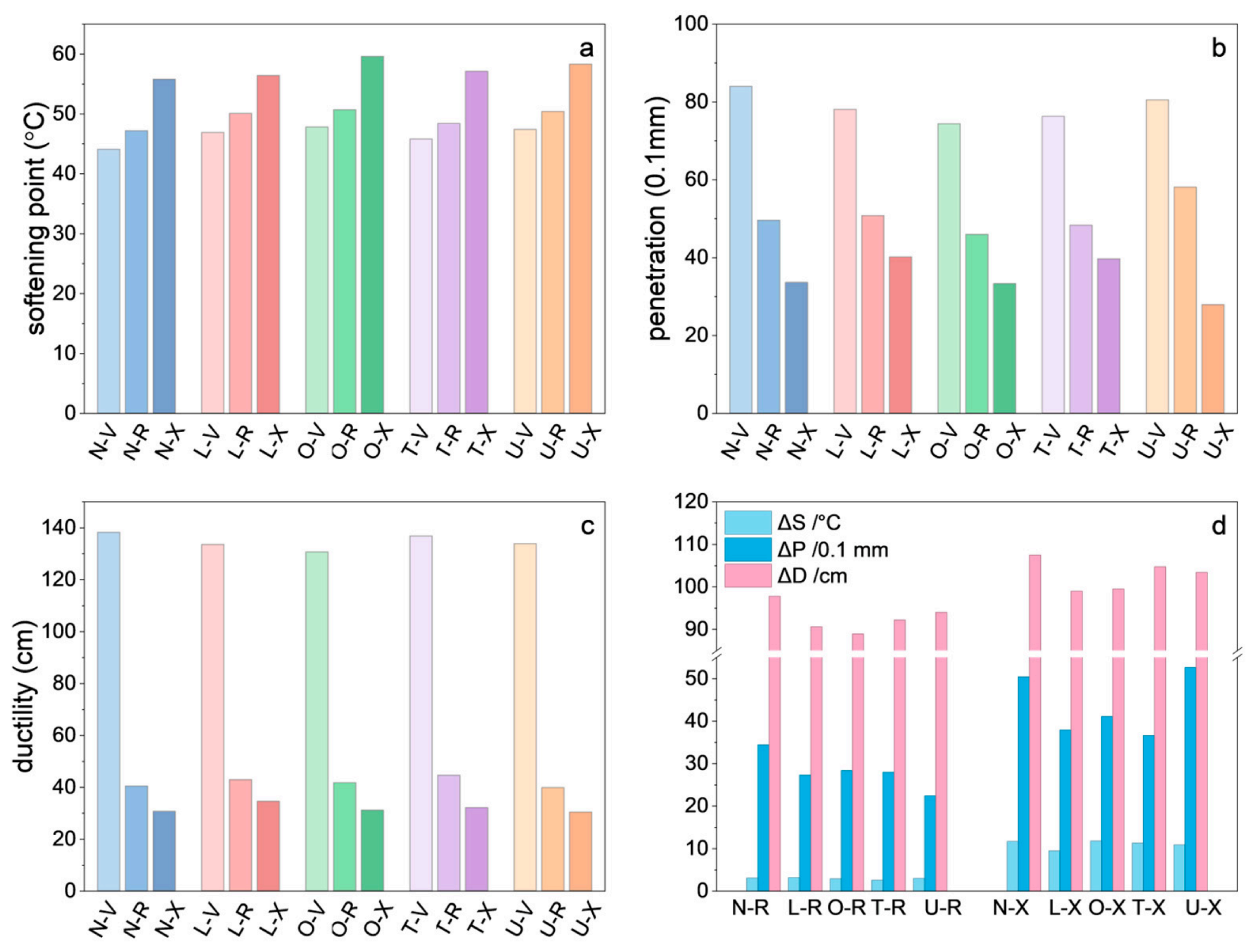
(**a**) Softening point, (**b**) penetration, (**c**) ductility of asphalt samples and (**d**) changes in conventional physical properties of asphalt after RTFOT and xenon-lamp ageing.

The addition of anti-aging agents slightly reduces the penetration and ductility of the unaged asphalt. Among these, the OMMT-modified asphalt exhibits the greatest reduction, which is ascribed to the dense network barrier formed by its nano-layered structure, restricting the mobility of the asphalt molecular chains [34,35,36]. After both short-term aging and xenon-lamp aging, the penetration and ductility of all modified asphalts continue to decrease, indicating progressive hardening of the asphalt due to aging (Figure 3a–c).

To further quantify the effect of anti-UV agents on long-term ageing, the absolute changes in softening point (ΔS), penetration (ΔP), and ductility (ΔD) of all asphalt samples after RTFOT and xenon-lamp ageing relative to their corresponding virgin states are calculated (Figure 3d). After xenon-lamp ageing, the modified asphalts generally showed smaller increases in softening point (except for OMMT) and smaller decreases in penetration (except for UV326) and ductility compared to neat asphalt. These results indicate that the anti-UV agents are generally effective in mitigating long-term UV ageing at the macroscopic level.

### 3.3. Evolution of Rheological Properties

**Figure 4:**
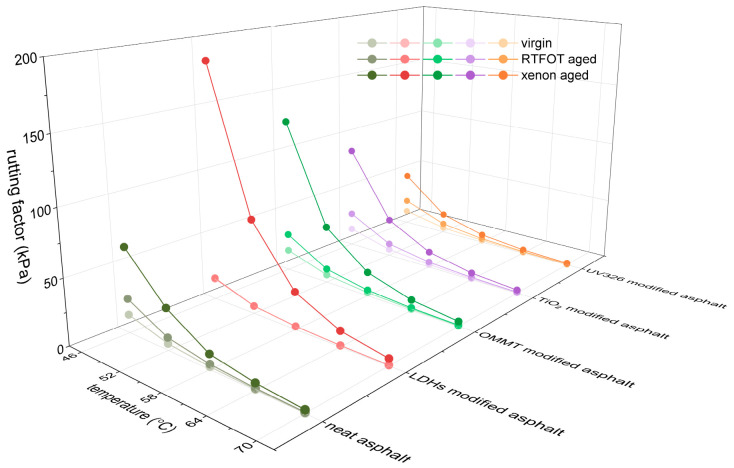
Rutting factor of asphalt samples.

**Figure 5:**
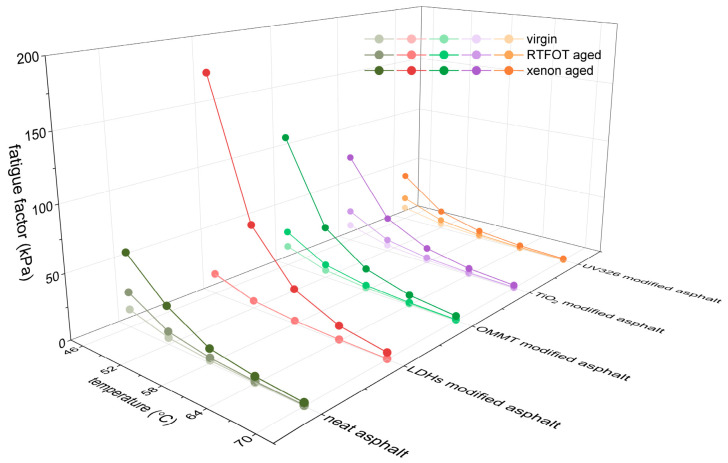
Fatigue factor of asphalt samples.

The DSR temperature sweep results (Figure 4 and Figure 5) illustrate the evolution of the high-temperature performance of various asphalt samples before and after aging. Aging increases the rutting factor (G*/sin δ) of all asphalts, indicating an enhancement in resistance to high-temperature deformation, albeit typically accompanied by a reduction in elasticity [37]. In both the unaged state and after RTFOT aging, the rutting factors of all modified asphalts remain essentially at the same level as that of the base asphalt, suggesting that the 3% additive dosage does not significantly affect the initial high-temperature performance or the resistance to short-term thermal aging. It should be noted that for the asphalt modified with LDHs, the rutting factor after short-term aging is nearly identical to that in the unaged state; consequently, the two curves almost overlap in Figure 4, as is also the case for the fatigue factor discussed later.

Following xenon-lamp aging, significant differences in high-temperature performance emerge among the asphalt samples. Notably, U-X exhibits a clear advantage within the 58–70 °C range, with its rutting factor even lower than that of the base asphalt. This indicates that UV326 effectively mitigates the loss in high-temperature rheological performance induced by UV aging. In contrast, L-X, O-X, and T-X demonstrate relatively higher rutting factors.

The variation trend of the fatigue factor (G*·sin δ) is similar to that of the rutting factor. The differences in fatigue factor among the unaged and RTFOT-aged asphalt samples are minimal. After xenon-lamp aging, L-X, O-X, and T-X all exhibit higher fatigue factors compared to the neat asphalt control sample, whereas U-X demonstrates the lowest fatigue factor. This result aligns with the evolution trend of the rutting factor, further confirming the superior ability of UV-absorbing additives in mitigating deep aging.

**Figure 6:**
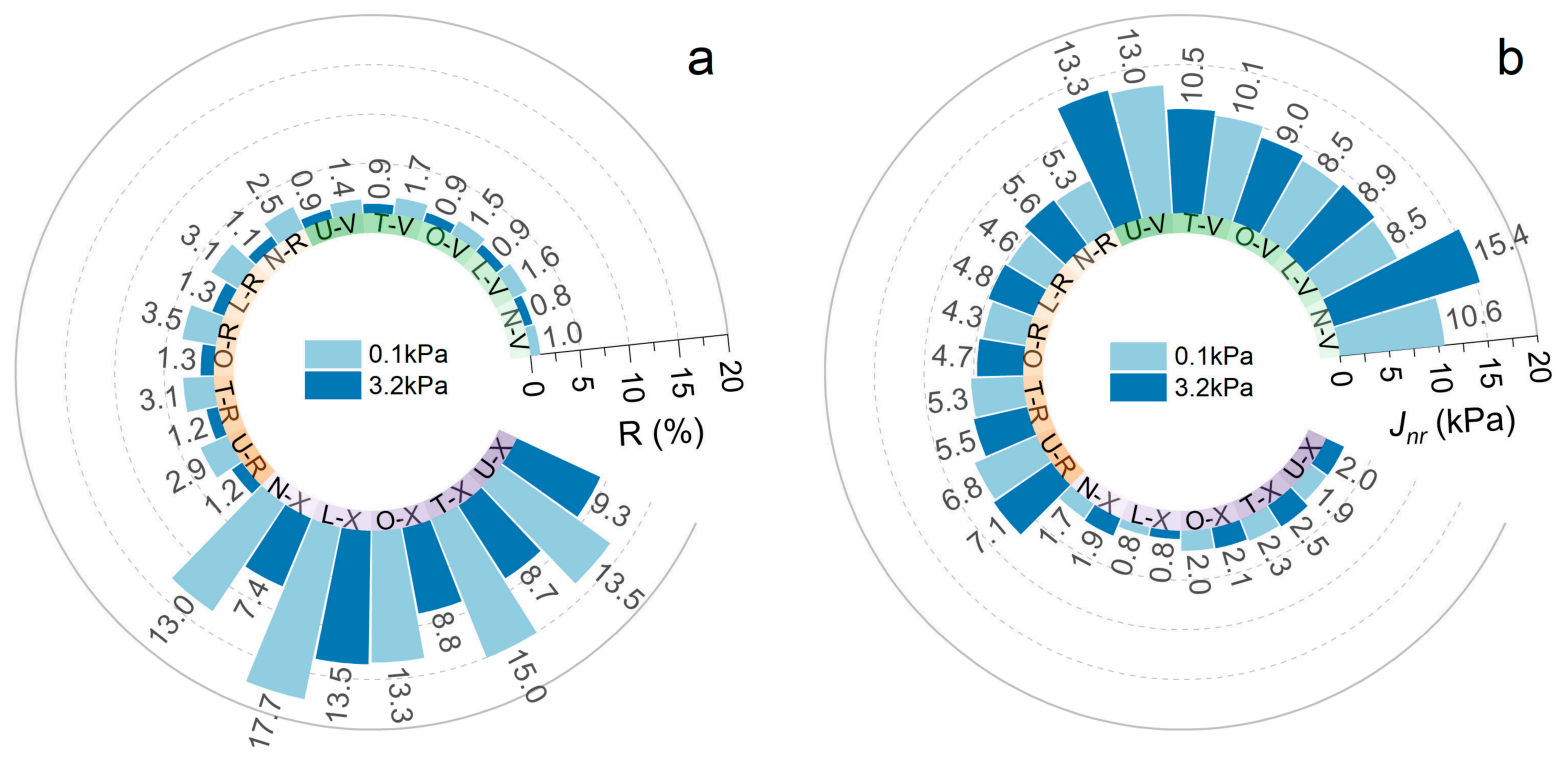
(**a**) *R* and (**b**) *J*_nr_ of asphalt samples.

The MSCR test results (Figure 6) indicate that the influence of UV anti-aging agents on the elastic recovery (*R*) and non-recoverable creep compliance (*J*_nr_) of asphalt depends primarily on the aging state, while also exhibiting somewhat complex evolutionary characteristics. In the unaged condition, all modified asphalts show higher *R* values than the base asphalt. Except for U-V, the *J*_nr_ values of modified asphalts prepared with other UV anti-aging agents are generally lower than those of the base asphalt. Compared with the short-term aged neat asphalt, modified asphalts under the same aging state—except for U-R—generally exhibit higher *R* and *J*_nr_ values. After xenon-lamp aging, all modified asphalts display higher *R* values than N-X; however, T-X, O-X, and U-X show higher *J*_nr_ than N-X, with only L-X having a lower *J*_nr_ value than N-X. The differential evolution of *R* and *J*_nr_ after xenon-lamp aging suggests that different anti-aging agents regulate the rheological properties of asphalt through distinct mechanisms.

**Figure 7:**
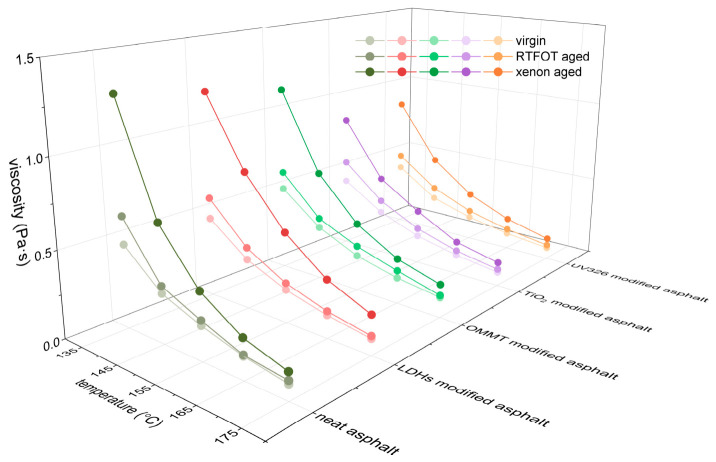
Viscosity of asphalt samples.

All UV anti-aging agents effectively suppressed the increase in viscosity of asphalt after xenon-lamp aging, with TiO_2_ and UV326 showing the most pronounced effects (Figure 7). Under unaged and short-term aged conditions, the viscosities of LDHs- and OMMT-modified asphalts were close to those of the neat asphalt at the corresponding aging states, while the viscosities of TiO_2_- and UV326-modified asphalts were slightly lower. After xenon-lamp aging, N-X, L-X, and O-X exhibited a significant rise in viscosity, whereas the increase for T-X and U-X was markedly smaller than that of the former three asphalt samples. This indicates that TiO_2_ and UV326 can more effectively inhibit the viscosity increase induced by UV aging.

### 3.4. Changes in Chemical Composition

**Figure 8:**
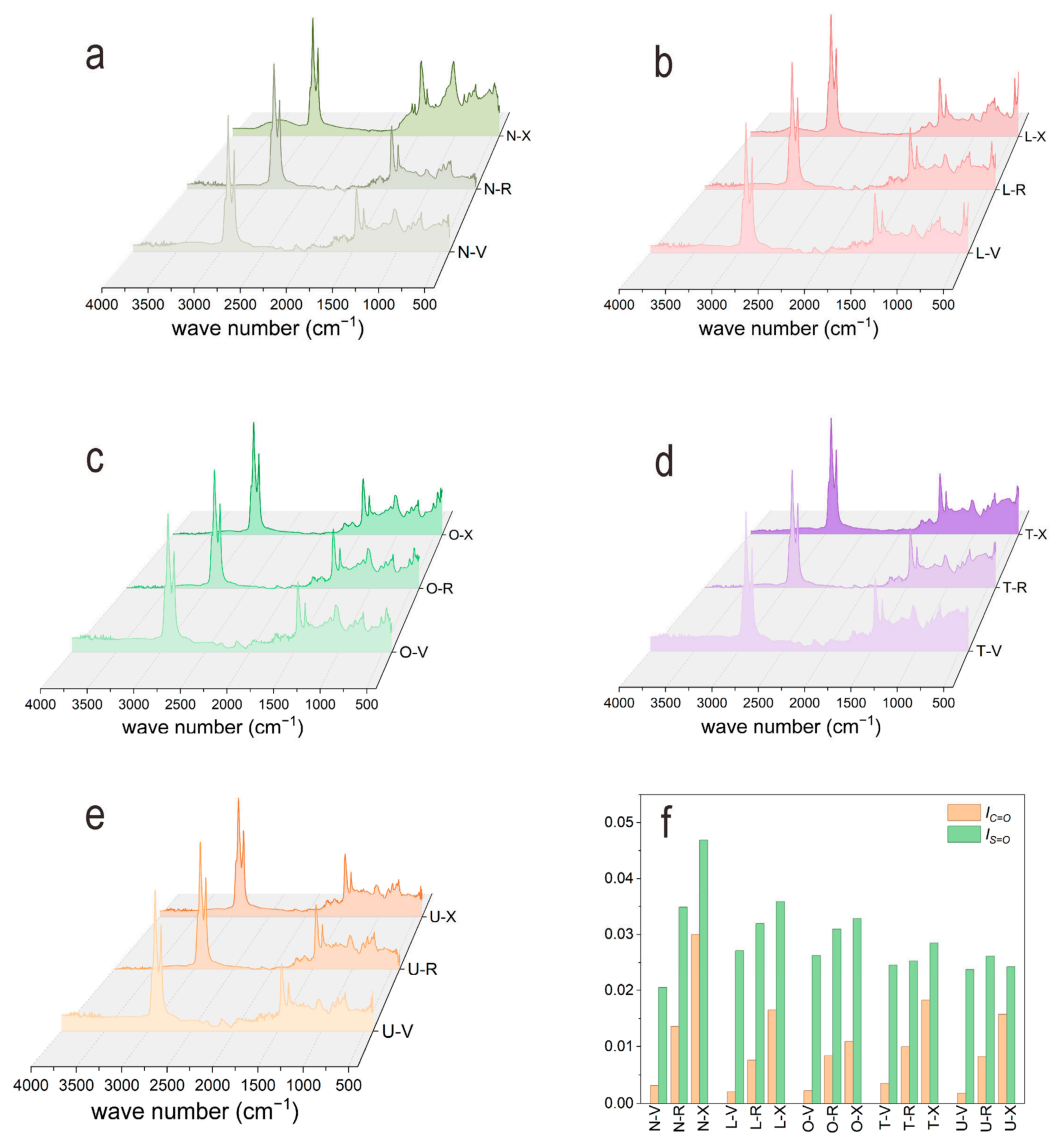
FTIR spectra of (**a**) neat asphalt, (**b**) LDH-modified, (**c**) OMMT-modified, (**d**) TiO_2_-modified, (**e**) UV326-modified asphalt samples. and (**f**) *I_C=O_* and *I_S=O_* of all asphalt samples.

FTIR analysis reveals that all four UV anti-aging agents effectively inhibit oxidation induced by ultraviolet irradiation. The calculated carbonyl index (*I_C=O_*) and sulfoxide index (*I_S=O_*) further quantify the ability of these agents to suppress asphalt oxidation. After RTFOT and xenon-lamp aging, the FTIR spectra of the modified asphalt samples showed no significant changes. In contrast, the neat asphalt after xenon-lamp aging exhibited a noticeable increase in infrared characteristic absorption at 1700 cm^−1^ (corresponding to carbonyl groups) and at 1030 cm^−1^ (corresponding to sulfoxide groups), indicating a substantial rise in carbonyl and sulfoxide content (Figure 8a–e). Based on *I_C=O_* and *I_S=O_* values, all four anti-aging agents significantly suppressed the formation of both carbonyl and sulfoxide groups, demonstrating broad-spectrum anti-aging performance. Among them, during xenon-lamp aging, OMMT showed the highest efficiency in inhibiting carbonyl formation, while UV326 exhibited the strongest suppression of sulfoxide generation (Figure 8f).

## 4. Discussion

To address the limitations of traditional single-index evaluations, this study employed PCA to reduce the dimensionality of multidimensional performance indicators, thereby achieving an objective and quantitative assessment of the performance of UV anti-aging agents. PCA is a statistical method that uses orthogonal transformation to convert a set of potentially correlated variables into linearly uncorrelated principal components. It can simplify a dataset while retaining its core information [38,39,40,41] and has been widely applied in the comprehensive performance evaluation of asphalt materials [42,43].

The effectiveness of PCA largely depends on the appropriate selection of input variables [44,45]. To ensure that the constructed PCA model accurately and consistently captures the performance evolution of asphalt samples during aging, the variable selection in this study followed three principles: representativeness, discriminative power, and non-redundancy.

(1) **Principle of Representativeness**: The selected variables should comprehensively cover the main performance dimensions of asphalt, including conventional physical properties, high-temperature rheological characteristics, and changes in chemical composition. This ensures that the PCA model can interpret aging behavior from multiple perspectives.
(2) **Principle of Discriminative Power**: For indicators that were measured under multiple temperature or stress conditions, the coefficient of variation (CV) was used to quantify the relative dispersion among asphalt samples at each condition. The condition with the highest CV was selected to maximize the indicator’s discriminatory power and avoid redundancy. For indices with a single measurement condition, the CV was not employed as a screening criterion; these were retained on the basis of their well-established engineering relevance to asphalt aging.
(3) **Principle of Non-redundancy**: To avoid multicollinearity and ensure model simplicity and interpretability, for indicators measured under multiple test conditions (e.g., rutting factor, viscosity), the condition with the highest CV was prioritized based on physical meaning and dispersion. This maximizes the information contribution of the indicator in the high-dimensional space.

Based on the above principles, ductility, penetration, softening point, *I_C=O_*, and *I_S=O_* were directly included in the PCA. For multi-condition indicators, the rutting factor and fatigue factor at 52 °C (highest CV), viscosity at 135 °C, *R*_3.2_ and *J*_nr3.2_ were selected (Table 3). The resulting input variable set thus comprised ten indices.

Furthermore, to ensure the validity and interpretability of the PCA model, the raw data were preprocessed prior to analysis:

(1) Directional Standardization of Indicators: For positive indicators (rutting factor, fatigue factor, *R*_3.2_, viscosity, *I_C=O_*, *I_S=O_*), the original data were retained. For negative indicators (ductility, penetration, and *J*_nr3.2_), a linear inversion by multiplying by –1 was applied to unify the directional interpretation, ensuring that an increase in value consistently represents a higher degree of asphalt aging.
(2) Data Normalization: Following directional standardization, Z-score normalization was applied to the data, setting the mean to zero and the standard deviation to one. This eliminates the influence of differing units and scales among indicators on the analytical results.

The preprocessed data were evaluated for suitability for PCA analysis using the Kaiser–Meyer–Olkin (KMO) test and Bartlett’s test of sphericity. The results (Table 4) show a KMO value greater than 0.5 and a significance level (*p*-value) for Bartlett’s test of less than 0.001, indicating that the dataset is appropriate for principal component analysis [46].

**Figure 9:**
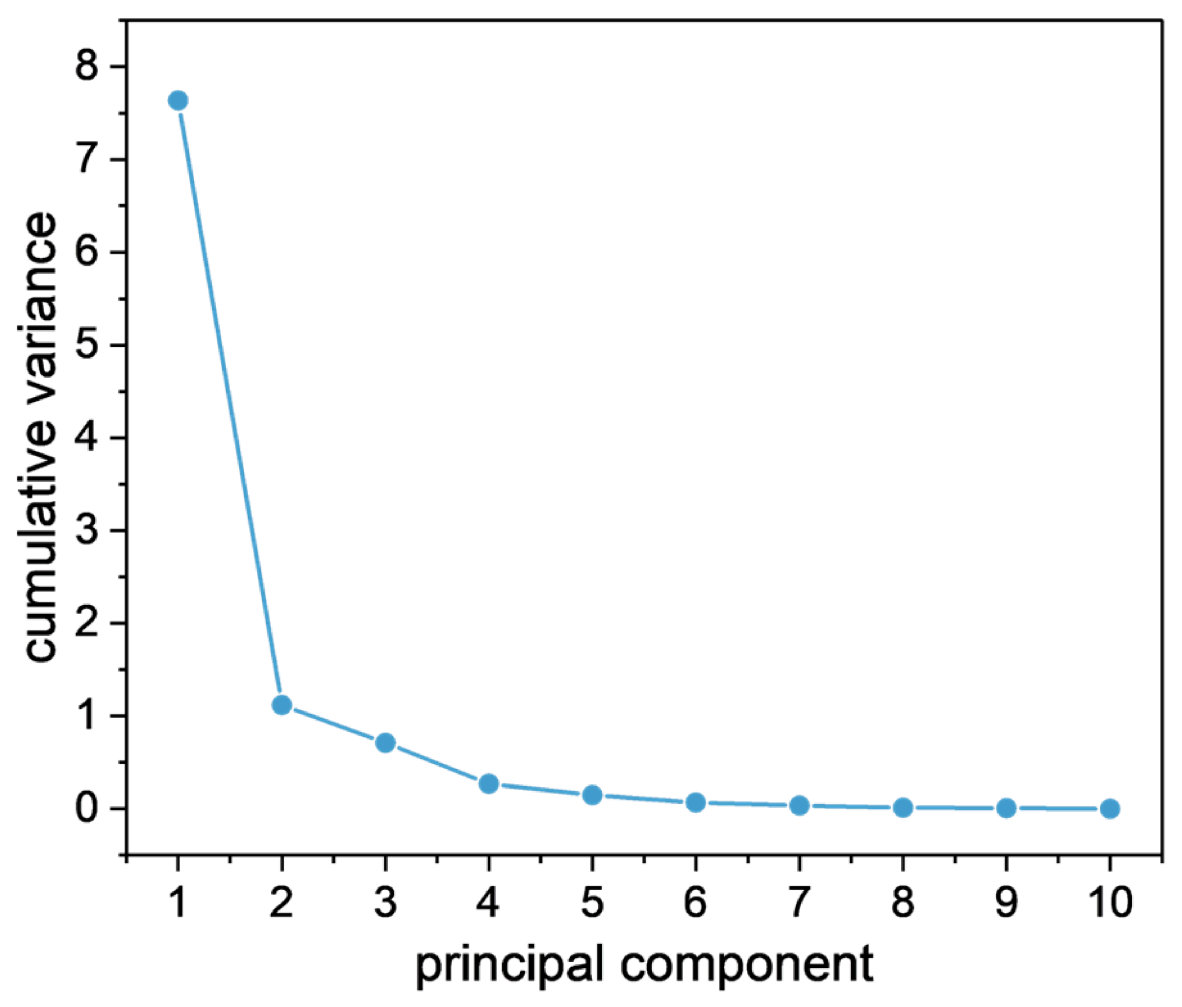
Scree plot of PCA.

Principal components (PCs) were extracted based on the criterion that the cumulative variance contribution rate should exceed 85%, ensuring sufficient representation of the original information. As shown in the total variance explained table (Table 5), extracting two principal components resulted in a cumulative variance contribution rate of 87.540%, with each of the first two PCs individually contributing more than 10% of the total variance. The scree plot (Figure 9) further revealed that the eigenvalue change stabilized after the second principal component. Consequently, two principal components were retained in this study for subsequent analysis.

Table 6 presents the loading matrix of different performance indicators on PC1 and PC2, offering key insights into the principal dimensions that characterize asphalt aging behavior.

All loading values for principal component PC1 fall within the range of 0.720–0.954, with a variance contribution rate of 76.373%, indicating that PC1 effectively integrates multidimensional aging-related information and can be interpreted as a comprehensive aging indicator. Among these, viscosity, *J*_nr3.2_, softening point and penetration exhibit the highest loadings, reaching 0.954, 0.921, 0.913, and 0.909 respectively, signifying their most significant contributions to PC1. It should be noted that since ductility, penetration, and *J*_nr3.2_ were inverted during preprocessing, their positive loadings indicate that a decrease in these indicator values correlates positively with an increase in PC1 scores—that is, asphalt aging severity increases with higher PC1 scores. Furthermore, the loadings for *R*_3.2_, fatigue factor, *I_C=O_* and rutting factor all exceed 0.85, further confirming that PC1 effectively captures the deterioration of both high- and low-temperature performance as well as chemical oxidation characteristics during aging. Notably, ductility and *I_S=O_*, after inversion, also show a relatively high loading of 0.822 and 0.720 respectively, demonstrating its role as an important evaluation indicator for comprehensive aging.

PC2 accounts for 11.167% of the variance, and its mixed positive and negative loading pattern reflects relative differences in the degradation rates of various performance indicators among asphalt samples during aging. Positive loadings on PC2 include *J*_nr3.2_, penetration, *I_C=O_*, ductility and *I_S=O_*, with values of 0.242, 0.329 0.283, 0.448, and 0.256 respectively, primarily representing aging-induced losses in low-temperature deformation capacity and increased susceptibility to cracking. Negative loadings on PC2 include viscosity, *R*_3.2_, fatigue factor and rutting factor with values of −0.132, −0.369, −0.468 and −0.477 respectively, mainly reflecting the deterioration of high-temperature performance. Therefore, an increase in PC2 score can be interpreted as a relatively faster degradation of low-temperature-related properties compared to high-temperature rheological performance, and vice versa. Notably, the softening point’s loading on PC2 is only –0.052, suggesting that it does not contribute to the second dimension, which primarily reflects the relative degradation rates of high- vs. low-temperature properties.

**Figure 10:**
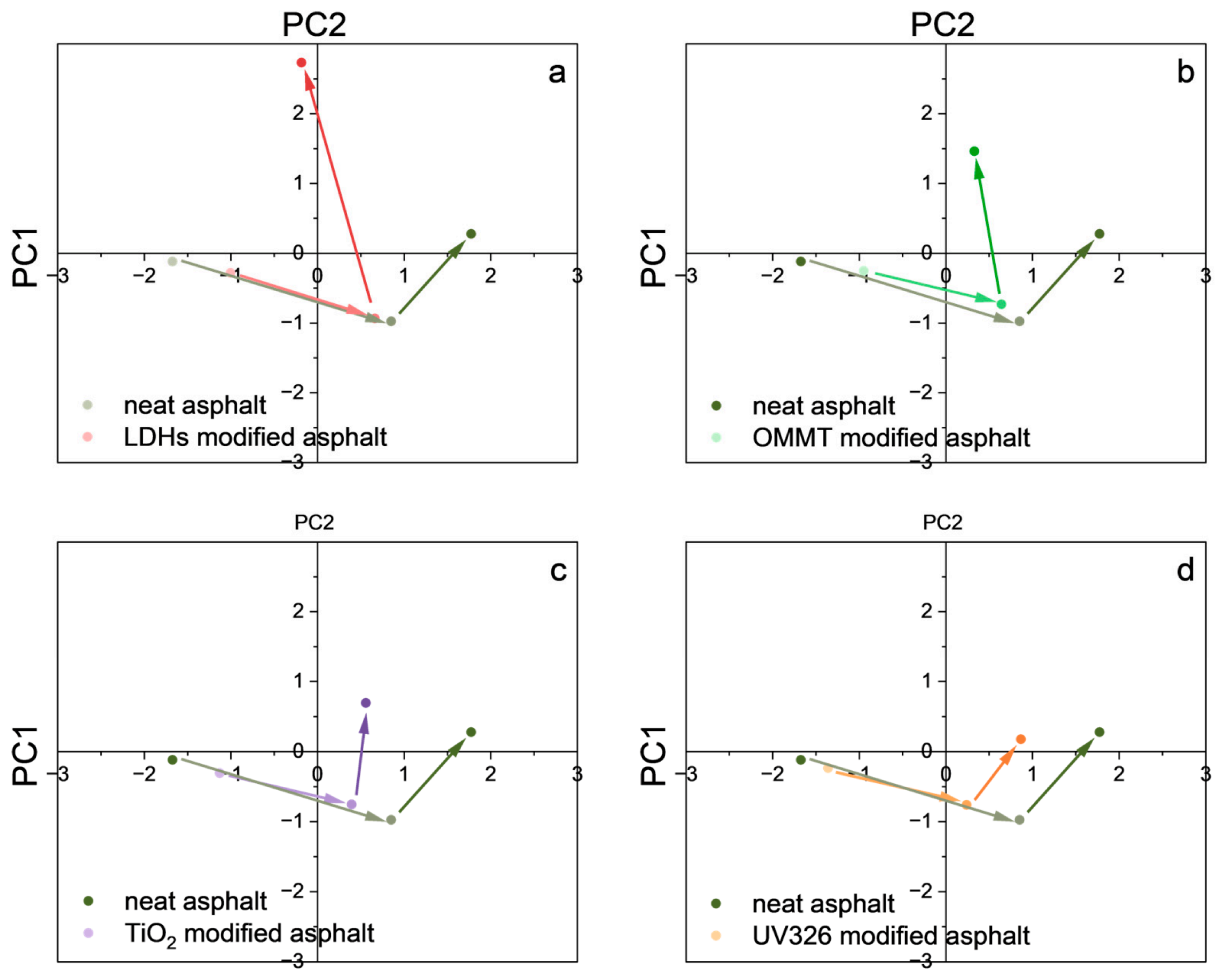
Aging trajectory quadrant diagram of (**a**) LDHs, (**b**) OMMT, (**c**) TiO_2_ and (**d**) UV326-modified asphalt.

Based on the principal component scores obtained from PCA for each sample (Table 7), an aging trajectory quadrant diagram (Figure 10) was constructed. The changes in PC1 and PC2 values during short-term aging (V–R) and UV aging (R–X) stages—ΔPC1, ΔPC2—along with the corresponding changes in trajectory vector angle (θ) were calculated (Table 8). This allows for a quantitative comparison of the effects of different UV anti-aging agents on the relative rates of high- and low-temperature performance degradation during asphalt aging.

During the V–R stage, the differences in θ angles among all asphalt samples were relatively small, indicating that UV anti-aging agents had minimal influence on the relative degradation rates of high- and low-temperature performance at this stage. Compared to low-temperature performance, the degradation rate of high-temperature rheological properties was slightly faster. Additionally, compared to neat asphalt, the magnitudes of ΔPC1 and ΔPC2 for each modified asphalt were smaller, combined with similar θ values, demonstrating a balanced retardation of aging during this stage.

In the R–X stage, the aging directions of different asphalt samples exhibited significant divergence, highlighting the differential effects of anti-aging agents:

- For neat asphalt, ΔPC1 and ΔPC2 were 0.92 and 1.25, respectively, with θ = 54°, indicating that xenon-lamp aging not only intensified the overall aging degree but also accelerated the degradation of low-temperature performance induced by UV aging.
- LDHs and OMMT, both acting as physical-shielding anti-UV agents, showed consistent aging paths: their ΔPC1 values were –0.85 and –0.31, respectively, reflecting effective suppression of overall aging, while ΔPC2 reached notably high values of 3.67 and 2.19, with very similar θ angles of −77° and −82°. This suggests that these two agents convergently retarded the loss of high-temperature rheological performance during this aging stage but failed to effectively inhibit the degradation of low-temperature properties.
- The light-reflective anti-UV agent TiO_2_ exhibited relatively low ΔPC1 and ΔPC2 values of 0.16 and 1.45, respectively, with θ = 84°, indicating that it slightly suppressed high-temperature performance degradation while delaying overall asphalt aging.
- The UV-absorbing anti-aging agent UV326 showed ΔPC1 and ΔPC2 values of 0.63 and 0.94, respectively, which were slightly lower than those of neat asphalt, and shared the same θ angle of 56°. Its PCA trajectory direction was closest to that of neat asphalt, suggesting that it delayed aging in a relatively balanced manner without significantly altering the relative progression of aging.

The PCA model, constructed based on the principles of representativeness, discriminative power, and non-redundancy, successfully reduced multiple performance indicators to two principal components: PC1 quantifies the overall aging degree, while PC2 reflects the relative degradation rates of high- and low-temperature performance. Dynamic trajectory analysis revealed that during the V–R stage, all asphalt samples displayed negative ΔPC2 values and similar vector angles, indicating that the relative degradation patterns of high- and low-temperature performance were highly similar between modified and base asphalts in this thermal-oxidative aging stage. In contrast, during the R–X stage, aging trajectories exhibited clear clustering characteristics: LDHs and OMMT simultaneously suppressed overall aging and high-temperature rheological loss, TiO_2_ primarily delayed the overall aging process while slightly affecting performance evolution, and UV326 maintained the existing relative degradation rates of high- and low-temperature performance while partially inhibiting aging. These distinctly clustered trajectory features demonstrate that anti-UV agents with the same protective mechanism exert similar influences on the degradation rates of asphalt high- and low-temperature performance induced by UV aging.

## 5. Conclusions

This study systematically investigated the regulatory effects of four UV anti-aging agents with three distinct mechanisms on asphalt aging behavior through multi-dimensional characterization and PCA. Based on a variable-selection strategy emphasizing representativeness, discriminative power, and non-redundancy, a stable and interpretable PCA model was established. This model reduced multiple correlated indicators to two principal components: PC1, representing the overall aging degree, and PC2, reflecting the relative degradation rates of high- and low-temperature performance.

PCA-based trajectory analysis revealed that during the V–R stage, all anti-aging agents exhibited certain retarding effects, reducing the increase in overall aging degree without altering the relative degradation rates between high- and low-temperature performance. In the R–X stage, however, different types of UV anti-aging agents showed significant differences in regulating the relative degradation rates: physical-shielding agents markedly suppressed the deterioration of high-temperature rheological performance but failed to effectively inhibit the rapid loss of low-temperature properties; the UV-absorbing agent delayed aging in a relatively balanced manner without substantially changing the inherent pattern of the aging process.

The integrated framework proposed in this study—combining multi-dimensional characterization, PCA-based dimensionality reduction, and analysis of relative performance degradation rates—provides a novel methodology for elucidating the differential impacts of anti-aging agents on asphalt aging behavior. The established connection between anti-aging mechanisms and performance-degradation patterns offers a scientific basis for the rational selection of anti-aging agents according to engineering performance requirements.

It should be noted that this work constitutes a preliminary comparative evaluation of anti-aging agents with different mechanisms. A specific base asphalt and four specific UV anti-aging agents were selected and modified at equal mass fractions to facilitate cross-comparison. Therefore, the conclusions drawn are valid only for the specific materials and dosages employed in this study. Given the distinct physicochemical properties of different additives and the fact that the dosage used is not necessarily their respective optimum efficiency point, the findings do not imply identical compatibility or effectiveness for other asphalt types or anti-UV agent contents. Future research should further optimize the optimal dosage of each additive to achieve more cost-effective engineering benefits.

Nevertheless, the framework proposed herein provides a new methodological foundation for the precise selection of anti-aging agents according to different protective mechanisms and engineering demands. Subsequent studies could incorporate time-series analysis or machine-learning algorithms to advance the model from state description to process prediction. By establishing quantitative relationships among aging time, aging conditions, and PCA scores, the performance of asphalt at any aging stage could ultimately be predicted.

## Figures and Tables

**Table 1:** Performance properties of base asphalt.

Performance		Value
density/g·cm^−3^		1.056
penetration (25 °C, 100 g, 5 s)/0.1 mm		84
ductility (15 °C)/cm		138.2
softening point/°C		44.1
flash point/°C		275
viscosity (60 °C)/Pa·s		167.5
after RTFOT	residual penetration ratio/%	59.0
	residual ductility/cm	40.4
	mass loss/%	0.2

**Table 2:** Sample naming convention.

	Neat Asphalt	LDHs-Modified	TiO_2_-Modified	OMMT-Modified	UV326-Modified
Unaged (Virgin)	N-V	L-V	T-V	O-V	U-V
Short-term (RTFOT)-aged	N-R	L-R	T-R	O-R	U-R
UV (Xenon)-aged	N-X	L-X	T-X	O-X	U-X

**Table 3:** CV for each indicator of asphalt samples.

Indicator		CV
Rutting factor	46 °C	106.7%
	52 °C	109.9%
	58 °C	108.0%
	64 °C	104.9%
	70 °C	101.7%
F Fatigue factor	46 °C	105.0%
	52 °C	105.4%
	58 °C	105.2%
	64 °C	103.3%
	70 °C	100.8%
*R*	0.1 kPa	96.9%
	3.2 kPa	110.9%
*J*_nr_	0.1 kPa	65.2%
	3.2 kPa	70.0%
Viscosity	135 °C	42.4%
	145 °C	41.5%
	155 °C	38.7%
	165 °C	35.9%
	175 °C	34.3%

**Table 4:** Value of KMO and Bartlett’s test of sphericity.

KMO	Bartlett’s Test of Sphericity (*p*)
0.739	<0.001

**Table 5:** Total variance explained.

PCs	Initial Eigenvalues			Sums of Squared Loadings		
	Total	Variance Percentage	Cumulative Variance Contribution Rate/%	Total	Variance Percentage	Cumulative Variance Contribution Rate/%
1	7.637	76.373	76.373	7.637	76.373	76.373
2	1.117	11.167	87.540	1.117	11.167	87.540
3	0.709	7.094	94.634			
4	0.269	2.689	97.323			
5	0.147	1.468	98.791			
6	0.068	0.681	99.472			
7	0.034	0.343	99.815			
8	0.013	0.127	99.942			
9	0.006	0.057	100			
10	4.287 × 10^−5^	0	100			

**Table 6:** Component loadings matrix of PC1 and PC2.

	Component Loadings Matrix	
	PC1	PC2
viscosity	0.954	−0.132
*J*_nr3.2_	0.921	0.242
softening point	0.913	−0.052
penetration	0.909	0.329
*R*_3.2_	0.896	−0.369
fatigue factor	0.864	−0.468
*I_C=O_*	0.861	0.283
rutting factor	0.858	−0.477
ductility	0.822	0.448
*I_S=O_*	0.720	0.256

**Table 7:** Principal component scores for each asphalt sample.

Samples	PC1	PC2
N-V	−1.67	−0.12
L-V	−1.00	−0.28
O-V	−0.95	−0.25
T-V	−1.13	−0.31
U-V	−1.36	−0.24
N-R	0.85	−0.97
L-R	0.66	−0.93
O-R	0.64	−0.73
T-R	0.39	−0.75
U-R	0.24	−0.76
N-X	1.77	0.28
L-X	−0.19	2.73
O-X	0.33	1.46
T-X	0.56	0.69
U-X	0.87	0.18

**Table 8:** Vector characteristics of the V–R and R–X stage for neat and modified asphalt.

Asphalt	V–R Stage			R–X Stage		
	ΔPC1	ΔPC2	*θ*/°	ΔPC1	ΔPC2	*θ*/°
neat asphalt	2.53	−0.85	−19	0.92	1.25	54
LDH-modified	1.67	−0.65	−21	−0.85	3.67	−77
OMMT-modified	1.59	−0.48	−17	−0.31	2.19	−82
TiO_2_-modified	1.52	−0.45	−16	0.16	1.45	84
UV326-modified	1.60	−0.53	−18	0.63	0.94	56

## Data Availability

The original contributions presented in this study are included in the article. Further inquiries can be directed to the corresponding authors.

## Appendix A

In the testing of asphalt properties, multiple replicate tests were conducted. The number of replicate tests and error control requirements for each indicator was implemented in accordance with the Chinese industry standard [47], as specified below:

Penetration tests were performed in triplicate. When the test result is less than 50 (0.1 mm), the allowable error is 2 (0.1 mm); when the test result is not less than 50 (0.1 mm), the relative allowable error is 4%.

Softening point tests were performed in duplicate, with an allowable error of 1 °C.

Ductility tests were performed in triplicate, with a relative allowable error of 20%.

Viscosity tests were performed in triplicate, with a relative allowable error of 3.5%.

DSR tests were performed in triplicate, with the relative allowable error as presented in Table A1.

**Table A1:** Relative allowable error for DSR tests.

Asphalt Properties		Relative Allowable Error/%
Unaged (Virgin)	G*/sin δ	5.2
Short-term (RTFOT) aged	G*/sin δ	8.4
UV (Xenon) aged	G*/sin δ	11.8

MSCR tests were performed in triplicate, with the relative allowable error as presented in Table A2.

**Table A2:** Relative allowable error for MSCR tests.

Asphalt Properties	Relative Allowable Error/%
*R*_0.1_	4.2
*R*_3.2_	4.7
*J*_nr0.1_	9.9
*J*_nr3.2_	10.0

## Appendix B

The choice of 3 wt% as a uniform comparative dosage was guided by two sets of preliminary experiments.

First, the anti-aging efficacy of LDHs was tested at 3% and 5% by weight under accelerated xenon-lamp irradiation (Figure A1). Without LDHs, the softening point of neat asphalt increased by 6.1°C after 96 h irradiation. With 3% LDHs, this increment was reduced to 2.0°C, and with 5% LDHs it was 1.5°C. The difference between the two dosages was only 0.5°C, indicating that LDHs already reach an efficacy plateau around 3%; a higher dosage provided no substantial additional benefit.

Second, the storage stability of LDH-modified asphalt was assessed at three concentrations (1%, 3%, and 5%) in three typical base asphalts by measuring the softening point difference between the top and bottom sections of a storage tube (Figure A2). Across all asphalt types and concentrations, the top-bottom softening point difference never exceeded 0.5°C, demonstrating that satisfactory dispersion and thermal storage stability are maintained even at 5%.

Together, these results confirm that 3 wt% LDHs lie well within both the effective plateau and the safe processing window, making this a technically sound and stable reference point for comparing the anti-UV aging mechanisms of different additives.
